# Herbal Medicines: Personal Use, Knowledge, Attitude, Dispensing Practice, and the Barriers among Community Pharmacists in Gondar, Northwest Ethiopia

**DOI:** 10.1155/2017/6480142

**Published:** 2017-08-22

**Authors:** Dessalegn Asmelashe Gelayee, Gashaw Binega Mekonnen, Seyfe Asrade Atnafe, Mequanent Kassa Birarra, Assefa Belay Asrie

**Affiliations:** ^1^Department of Pharmacology, College of Medicine and Health Sciences, University of Gondar, Gondar, Ethiopia; ^2^Department of Clinical Pharmacy, College of Medicine and Health Sciences, University of Gondar, Gondar, Ethiopia

## Abstract

**Background:**

Herbal medicine use is increasing and the global market is estimated to be US$107 billion by the year 2017.

**Objectives:**

This study aimed at assessing community pharmacists' personal use, knowledge, attitude, dispensing practice, and the barriers regarding herbal medicines.

**Methods:**

Institution based cross-sectional study was conducted among 47 community pharmacists in Gondar, Northwest Ethiopia, using a structured interviewing questionnaire.

**Results:**

Nearly half of the respondents (*n* = 22, 46.8%) sometimes use herbal medicines. Although knowledge related to such preparations was self-rated as poor/acceptable (*n* = 34, 72.4%), majority (*n* = 44, 93.7%) of community pharmacists agree/strongly agree that herbal medicines have beneficial effects. Only 6 (12.7%) of them are sometimes/often engaged in dispensing herbal medicines and most of them (*n* = 34, 72.3%) rarely/never counseled clients regarding these preparations. Limited knowledge on and access to information regarding herbal medicines are the main barriers to the pharmacists' practice.

**Conclusion:**

Although community pharmacists in Gondar, Northwest Ethiopia, commonly use and demonstrated good attitude towards herbal medicines, they are less involved in dispensing such products. They are also challenged with limited knowledge on and access to herbal medicine information. Thus, pharmacy educators, professional organizations, and the government shall pay more attention to solve the problem. Regulatory provisions on herbal medicine dispensing must be enacted and communicated very well.

## 1. Introduction

The use of herbal medicines in healthcare practice is increasing and the global market for such products is estimated to be US$107 billion by the year 2017 [1]. However, there is also a growing concern on safety of these preparations [2] due to lack of suitable quality controls, inadequate labeling, and the absence of appropriate patient information among others [3]. In United States alone, an estimated 2000 hospitalizations took place each year as a result of ingestion of dietary supplements [4]. Nowadays, consumers' demand on herbal medicine information is very high and pharmacists, if knowledgeable, are in an ideal position to address this demand. They are the third largest regulated healthcare professional groups in the world [5]. A 2012 report conducted in 90 countries stated that 55% of pharmacists were working in community pharmacies [6].

About 80% of the human population in Ethiopia is said to be dependent on traditional medicine for primary healthcare services and most of this comes from plants [7]. Moreover, prevalence of chronic illnesses in the nation is increasing [8] and previous studies showed that the use of herbal remedies among such patients is very high [9]. Thus, pharmacists in Ethiopia are required more than ever to improve their role as experts of herbal medicines and in providing appropriate information to clients. Consumers in general should be well informed about herbal medicine use, side effects, and medicine interaction and be closely monitored to achieve the therapeutic outcomes. However, there is no any regulation on the sale of herbal medicines in Ethiopia [10] and the curriculum for undergraduate pharmacy program in the nation regarding herbal medicine seems to be inadequate. It only comprises 2-credit hour course on “Alternative and Complementary Medicine,” 4-credit hour course on “Pharmacognosy,” and 3-credit hour course on “Chemistry of Natural Products” [11]. Previous studies demonstrated that such courses in the curriculum alone were perceived to be inadequate to prepare pharmacists as experts of herbal medicines. [12–14]. This study was therefore intended to assess the personal use, knowledge, attitude, dispensing practice, and the barriers regarding herbal medicines in a low-income country setting. The findings will support the design of educational programs and objectives by pharmacy schools and professional organizations.

## 2. Methods

This cross-sectional study was conducted among pharmacists working in community pharmacies of Gondar Town, Northwest Ethiopia, from October 2016 to January 2017. Fifty-three medication retail outlets (19 pharmacies and 34 medicine stores) were registered in the town in 2014. In the nation, pharmacies are run by pharmacists and medicine stores by pharmacy technicians. Community pharmacist, in this study, refers to both pharmacy technicians and pharmacists and community pharmacy refers to both medicine stores and pharmacies. Data were collected by the authors during the day time working hours and mostly in the morning. Respondents were interviewed using a structured questionnaire that was adapted from previous studies [15, 16] with some modifications and is attached as a supplementary material (in Supplementary Material available online at https://doi.org/10.1155/2017/6480142). It consists of 5 parts: Part 1: 7 questions related to sociodemography with open and closed questions; Part 2: 4 questions on practice related to herbal medicines with 5-point Likert scale responses (1 = never, 2 = rarely, 3 = sometimes, 4 = often, and 5 = always); Part 3: 5 questions related to attitude towards herbal medicines with 4-point Likert scale responses (1 = strongly disagree, 2 = disagree, 3 = agree, and 4 = strongly agree); Part 4: 4 questions on knowledge related to herbal medicines with 4-point Likert scale responses (1 = poor, 2 = acceptable, 3 = good, and 4 = very good); and Part 5: 4 miscellaneous questions of yes/no types related to practice and barriers in dispensing herbal medicines.

The questionnaire was pretested on 5 pharmacy technicians working as part time in private pharmacies and modifications were made before the actual data collection. It was also evaluated and approved for face validity by three senior pharmacists who are academicians and researchers. The reliability assessment of the different subcomponents of the questionnaire after data collection revealed a Cronbach's alpha value of 0.713 for practice (4 items), 0.857 for attitude (5 items), and 0.872 for knowledge (4 items).

All community pharmacies in the town were approached in the study and about 47 pharmacists were willing to participate. The collected data were cleared, entered into, and analyzed by using the Statistical Package for Social Sciences (SPSS) version 20.0 for windows (SPSS Inc., Chicago, IL). The results were described in terms of frequencies, percentages, and means. Mann–Whitney *U* test was employed to test group differences (based on sociodemography) on Likert scale responses of practice, attitude, and knowledge related to herbal medicines. Pearson's Chi-square test of independence and Fisher's exact test were used to assess association between sociodemographic factors and miscellaneous questions included in the questionnaire. In both tests, *P* value < 0.05 was considered significant. An ethical clearance was taken from the school of Pharmacy, University of Gondar (SOP1018/2009), and all respondents were asked for their consent before participation in the study. Personal information was deidentified prior to the data analysis.

## 3. Results

### 3.1. Sociodemographic Characteristics

This survey was conducted among 47 pharmacists working in community pharmacies located in Gondar Town and all of them responded. The majority were male (*n* = 31, 66%), in the 23–28 years age interval (*n* = 25, 53.2%), at least B. Pharm degree holder (*n* = 27, 57.4%) had work experience of 4 years and below (*n* = 25, 53.2%) and were employee (*n* = 28, 59.6%). Most of the respondents (*n* = 42, 89.4%) did not attend any additional training on herbal medicines and 36 (76.6%) stated having limited access to information on herbal medicines. Pharmacists were perceived to be unauthorized to dispense herbal medicines by most (*n* = 33, 70.2%) respondents. Majority of them (*n* = 30, 63.8%) reported a sale of herbal medicines in nonpharmacy settings such as in supermarkets by a laypersons and this was regarded to be inappropriate by 43 (91.5%) participants, Table 1.

Females were more likely than males to opine that they have easy access to information regarding herbal medicines (Fisher's exact test *P* < 0.001) as well as observing that herbal medicines are being dispensed outside pharmacies such as in shops (*X*^2^ = 5.797, df = 1, *P* = 0.016). Those more experienced also were more likely than their counter groups to witness that herbal medicines are being dispensed outside pharmacies (*X*^2^ = 5.797, df = 1, *P* = 0.016).

### 3.2. Practice Related to Herbal Medicines

As shown in Table 2, the community pharmacists commonly use herbal medicines. Nearly half of the pharmacists (*n* = 22, 46.8%) sometimes use herbal medicines for self-treatment yet only 6 (12.7%) reported to sometimes/often dispense these preparations. Most of the respondents (*n* = 34, 72.3%) never/rarely counseled clients regarding herbal medicines and only 11 (23.4%) pharmacists have ever received inquiries on such preparations.

Sex based difference was observed on counseling customers on herbal medicines (females: *N* = 16, mean rank = 30.53; males: *N* = 31, mean rank = 20.63; *U* = 143.5, *P* = 0.013). Thus, females reported to be more engaged than males in counseling their clients regarding herbal medicines. Those more experienced in the profession more frequently received herbal medicine related inquiries compared to the less experienced ones (work experience of 1–4 years: *N* = 25, mean rank = 20.42; work experience of 5–16 years: *N* = 22, mean rank = 28.07; *U* = 185.5, *P* = 0.041).

### 3.3. Attitude towards Herbal Medicines

About 44 (93.7%) respondents agree/strongly agree that herbal medicines have beneficial effects. However, placebo effect from such preparations was also opined by 33 (70.2%) of them. While 24 (51.1%) pharmacists disagree/strongly disagree that herbal medicines have less side effects than conventional medicines, 38 (80.9%) agree/strongly agree that herbal medicines have significant interactions with conventional medicines. In addition, majority (*n* = 35, 74.5%) disagree/strongly disagree that herbal medicines are sufficiently studied (Table 3).

Sex, age, educational level, work experience, and pharmacy ownership have influenced the attitude of respondents on herbal medicines. Accordingly females believe more than males that herbal medicines are sufficiently studied (*P* = 0.006). Those in the age range of 23–28 years, diploma holders, and the employee pharmacists were more likely to agree than their counter groups that herbal medicines have significant interactions with conventional medicines with respective *P* values of 0.024, 0.035, and 0.045. Those more experienced were more likely to suggest that herbal medicines have placebo effects than their counter group (*P* = 0.030).

### 3.4. Knowledge Related to Herbal Medicines

Pharmacists in this study were asked to self-rate their knowledge about herbal medicines. Accordingly, the majority (*n* = 21, 44.7%) rated their knowledge acceptable, while 13 (27.7%) rated it poor. About 25 (53.2%), 23 (*n* = 48.9%), and 24 (51.1%) respondents described that their knowledge on herbal medicine interaction, herbal medicine side effects, and precautions is poor.  Table 4.

Knowledge related to herbal medicines was observed to vary based on work experience and sex of respondents. Thus, females were more likely than males to better rate their knowledge on herbal medicines in general (*P* < 0.001), knowledge about herbal medicine interactions (*P* = 0.000), knowledge about herbal medicine side effects (*P* = 0.026), and knowledge about herbal medicine precautions (*P* = 0.008). Similarly the less experienced pharmacists were more likely to better rate their knowledge than the more experienced about herbal medicine interactions (*P* = 0.021), knowledge about herbal medicine side effects (*P* = 0.037), and knowledge about herbal medicine precautions (*P* = 0.031).

## 4. Discussion

The present study demonstrated that herbal medicines are commonly used by community pharmacists in Gondar Town which is similar to other studies [17–21]. This signifies an increasing acceptance of herbal medicines among the healthcare professionals. However, respondents in the present study were less involved in dispensing these preparations. Several factors might be accounted for this. Most of the respondents perceived that pharmacists are not currently authorized to dispense such remedies, did not receive any additional training related to such preparations, and have limited access to information regarding herbal medicines. In fact there are no regulatory provisions regarding the sale of such preparations in Ethiopia [10] though there is high use of herbal remedies in the nation [7]. Limited access of information related to herbal medicines is also identified as a barrier to the practice of pharmacists in a Saudi study by Al-Arifi [16]. In the present study, males were more likely than females to have limited access to such information. Inadequacy of curricular training on herbal medicines was also reported in a Nigerian study among hospital pharmacists [22].

Most respondents reported the sale of herbal medicines in settings other than pharmacies and almost all discouraged the practice. This is acceptable since these remedies are not without adverse effects and medicine interactions [23] and thus it is better to dispense them by pharmacists than by a layperson. Although pharmacists worldwide are said to be very accessible professionals [5, 6], counseling practice of respondents on herbal medicines is very minimal and majority of them never/rarely received inquiries related to herbal medicines. This might be because of their limited involvement in dispensing these preparations and limited knowledge regarding herbal preparations. Majority of respondents claimed to have acceptable general knowledge about herbal medicines and more than a quarter of them described their knowledge as poor. In other studies as well [24–26], inadequate knowledge of herbal medicines is reported as a barrier to the practice of pharmacists. In the present study, females reported to be more engaged than males in counseling their clients regarding herbal medicines and they tend to believe more than males that these preparations are sufficiently studied. Those more experienced in the profession receive herbal medicine related inquiries more frequently compared to the less experienced ones.

In an attempt to improve evidence-based practice among pharmacists and physicians, developing and evaluating a formulary of herbal medicinal products available in local pharmacies were done in Malta. The finding was improved quality, evidence-based prescribing together with enhanced monitoring and improved patient care [27]. Therefore, similar interventions may be appropriate in Ethiopia as well.

Nearly half of respondents described that their knowledge on herbal medicine interaction, herbal medicine side effects, and precautions is poor. This finding strengthens the report by Oshikoya et al. that pharmacists in Nigeria also exhibited poor knowledge with regard to the indications, contraindications, and safety profiles of these remedies [24]. In the present study, female gender and less work experience were associated with higher self-rating of knowledge regarding herbal medicines.

Data regarding pharmacists' use, dispensing practice, attitude, and knowledge related to herbal medicines in Ethiopia is very rare. In this regard, the findings of the present study imply that community pharmacists often use these remedies personally and have good attitude. But they are less involved in the sale of these remedies and counseling clients. The main barriers were lack of knowledge, limited access of information, and unregulated sale of the preparations in the nation.

This study is not without limitation. Due to small sample size, it may not be generalized to community pharmacists in the nation, and knowledge should be better assessed through questions specific to the use, safety, and interaction of herbal medicines.

## 5. Conclusion

Community pharmacists in Gondar, Northwest Ethiopia, commonly use herbal medicines and demonstrated good attitude. However, there is a need for additional training and improved access to reliable sources of information if they are to be engaged in provision of pharmaceutical care service related to herbal medicines. Pharmacy schools need to reconsider curricular changes to improve the pharmacists' knowledge of these remedies and continuous professional development approaches may also be important.

## Supplementary Material

This questionnaire was designed to evaluate personal use of herbal medicines, knowledge, attitude, dispensing practice and the barriers among community pharmacists in Ethiopia.

## Figures and Tables

**Table 1:** Demographic characteristics (*N* = 47).

Variable	*n* (%)
Sex	
Female	16 (34%)
Male	31 (66%)
Age (year) mean ± SD = 30.7 ± 6.9	
23–28 years	25 (53.2%)
29–51 years	22 (46.8%)
Educational level	
Diploma	20 (42.6%)
B. Pharm	26 (55.3%)
M.S.	1 (2.1%)
Work experience in community pharmacy (years) 5.3 ± 3.7 (mean ± SD)	
1–4	25 (53.2%)
5–16	22 (46.8%)
Additional work experience	
Yes	22 (46.8%)
No	25 (53.2%)
Pharmacy ownership	
Owner	19 (40.4%)
Employee	28 (59.6%)
Attend additional training on herbal medicines	
Yes	5 (10.6%)
No	42 (89.4%)
Easily access information regarding herbal medicines	
Yes	11 (23.4%)
No	36 (76.6%)
Pharmacists are authorized to dispense herbal medicines	
Yes	14 (29.8%)
No	33 (70.2%)
Observed herbal medicines being dispensed outside pharmacies such as in supermarkets	
Yes	30 (63.8%)
No	17 (36.2%)
Appropriate to dispense herbal medicines by any person	
Yes	4 (8.5%)
No	43 (91.5%)

**Table 2:** Practice related to herbal medicines (*N* = 47).

Practice	Response *n* (%)				
	Never	Rarely	Sometimes	Often	Always
Dispense herbal medicines in the pharmacy	26 (55.3)	15 (31.9)	5 (10.6)	1 (2.1)	0
Use herbal medicines for self-treatment	13 (27.7)	12 (25.5)	22 (46.8)	0	0
Counsel customers about using of herbal medicines	18 (38.3)	16 (34.0)	12 (25.5)	0	1 (2.1)
Received inquiries related to herbal medicines	21 (44.7)	15 (31.9)	10 (21.3)	1 (2.1)	0

5-point Likert scale (1 = never, 5 = always).

**Table 3:** Attitude towards herbal medicines (*N* = 47).

Attitude towards herbal medicines	Response *n* (%)			
	Strongly disagree	Disagree	Agree	Strongly agree
Herbal medicines have beneficial effect	1 (2.1)	2 (4.2)	31 (66)	13 (27.7)
Herbal medicines have fewer side effects than conventional medicines	4 (8.5)	20 (42.6)	19 (40.4)	4 (8.5)
Herbal medicines have placebo effect	2 (4.3)	12 (25.5)	30 (63.8)	3 (6.4)
Herbal medicines are sufficiently studied	18 (38.3)	17 (36.2)	10 (21.3)	2 (4.2)
Herbal medicines have significant interactions with conventional medicines	1 (2.1)	8 (17.0)	32 (68.1)	6 (12.8)

4-point Likert scale (1 = strongly disagree, 4 = strongly agree).

**Table 4:** Knowledge related to herbal medicines (*N* = 47).

Pharmacist's self-rating for knowledge related to herbal medicines	Response *n* (%)			
	Poor	Acceptable	Good	Very good
Knowledge about herbal medicines in general	13 (27.7)	21 (44.7)	12 (25.5)	1 (2.1)
Knowledge about herbal medicine interactions	25 (53.2)	12 (25.5)	10 (21.3)	0
Knowledge about herbal medicine side effects	23 (48.9)	15 (31.9)	7 (14.9)	2 (4.3)
Knowledge about herbal medicine precautions	24 (51.1)	16 (34.0)	5 (10.6)	2 (4.3)

4-point Likert scale (1 = poor, 4 = very good).
