# Supplementary material for: Herbal Medicines: Personal Use, Knowledge, Attitude, Dispensing Practice, and the Barriers among Community Pharmacists in Gondar, Northwest Ethiopia
Source: Evid Based Complement Alternat Med. 2017 Aug 22;2017:6480142. doi: 10.1155/2017/6480142 (PMC5585575; doi:10.1155/2017/6480142)
Supplement: Supplementary file 1 — This questionnaire was designed to evaluate personal use of herbal medicines, knowledge, attitude, dispensing practice and the barriers among community pharmacists in Ethiopia. [file 6480142.f1.doc]

**Herbal Medicines: Personal use, knowledge, attitude, dispensing practice and the barriers among community pharmacists in Gondar, Northwest Ethiopia**

Part I: Socio-demography

1. Sex: female male
2. Age (yr):________
3. Level of education: diploma B.Pharm MSc
4. Experience in community pharmacy (yr):____________
5. Employment status: employee owner
6. Additional work experience? yes no
7. Did you attend any training related to herbal drugs? yes no

**Part II: Practice related to herbal drugs**

| **Practice** | **Response** | | | | |
| --- | --- | --- | --- | --- | --- |
|  | **Never** | **Rarely** | **Sometimes** | **Often** | **Always** |
| 1. Do you dispense herbal drugs in your pharmacy? |  |  |  |  |  |
| 1. Do you use herbal drugs for self treatment? |  |  |  |  |  |
| 1. Do you counsel your customers about using of herbal drugs? |  |  |  |  |  |
| 1. Do you get inquiries related to herbal drugs? |  |  |  |  |  |

**Part III: Attitude towards herbal drugs**

| **Attitude** | **Response** | | | |
| --- | --- | --- | --- | --- |
|  | **SD** | **D** | **A** | **SA** |
| 1. Do you agree that herbal drugs have beneficial effect? |  |  |  |  |
| 1. Do you agree that herbal drugs have fewer side effects than conventional medicines? |  |  |  |  |
| 1. Do you agree that herbal drugs have placebo effect? |  |  |  |  |
| 1. Do you agree that herbal drugs are sufficiently studied? |  |  |  |  |
| 1. Do you agree that herbal drugs have significant interactions with conventional medicines? |  |  |  |  |
| **SD=strongly disagree;D=disagree;A=agree; SA=strongly agree** | | | | |

**Part IV:Knowledge about herbal drugs**

| **knowledge** | **Response** | | | |
| --- | --- | --- | --- | --- |
|  | **Very good** | **Good** | **Acceptable** | **Poor** |
| 1. How do you rate your knowledge about herbal drugs in general? |  |  |  |  |
| 1. How do you rate your knowledge about herbal drug interactions? |  |  |  |  |
| 1. How do you rate your knowledge about herbal drug side effects? |  |  |  |  |
| 1. How do you rate your knowledge about herbal drug precautions? |  |  |  |  |

**PartV: Miscellaneous questions**

1. Do you easily access information regarding herbal drugs? yes no
2. Do you think pharmacies are authorized to dispense herbal drugs? yes no
3. Do you know herbal drugs are dispensed outside pharmacies such as in shops? yes no
4. Do you think it is appropriate for herbal drugs to be dispensed by any person? yes no
